# Comparative analysis and phylogeny of mitochondrial genomes of Pentatomidae (Hemiptera: Pentatomoidea)

**DOI:** 10.3389/fgene.2022.1045193

**Published:** 2022-11-11

**Authors:** Dan Lian, Jiufeng Wei, Chao Chen, Minmin Niu, Hufang Zhang, Qing Zhao

**Affiliations:** ^1^ College of Plant Protection, Shanxi Agricultural University, Taigu, China; ^2^ Department of Biology, Xinzhou Teachers University, Xinzhou, China

**Keywords:** Pentatomidae, mitochondrial genomes, Phyllocephalini, phylogenetic analysis, *Chalcopis*, *Gonopsimorpha*, *Gonopsis*

## Abstract

The Phyllocephalini is a group of herbivorous insects in Pentatomidae, which lack distinctive morphological characteristics and systematic studies. Up to now, there are only two complete mitochondrial genomes of Phyllocephalini have been reported. In this study, we sequenced and analyzed the complete mitochondrial genomes of three Phyllocephalini species, *Gonopsis coccinea*, *Gonopsimorpha nigrosignata,* and *Chalcopis glandulosus,* which were 16,534, 16,531, and 16,534 bp in length, respectively. The mitochondrial genomes contained 37 genes, including 13 protein-coding genes, two rRNA genes, 22 tRNA genes, and a control region. The gene arrangement was consistent with that of the putative ancestral insect, with no rearrangement. The *cox1* gene of Pentatomidae showed the lowest evolutionary rate among the protein-coding genes, the mean genetic distance of species, genera, and subfamilies of Pentatomidae increased hierarchically based on *cox1* gene. The *16S rRNA* of Pentatomidae was more conserved than *12S rRNA* in sequence and secondary structure. All tRNAs could be folded into a typical cloverleaf structure except *trnS1*. The stem region was more conserved than the loop region in the secondary structure of tRNAs within Pentatomidae. *Gonopsis coccinea* and *Gonopsimorpha nigrosignata* had one type of tandem repetition unit in the control region, while *C. glandulosus* had two types. The heterogeneity analysis of Pentatomidae showed that Phyllocephalinae was the most heterogeneous. Phylogenetic trees based on the newly obtain mitochondrial genomes along with other 50 mitochondrial genomes of Pentatomidae using Bayesian Inference and Maximum Likelihood strongly supported the following three relationships: (((*Anaxilaus* + (*Plautia* + *Glaucias*)) + (*Nezara* + *Palomena*)) + (Eysarcorini + Carpocorini)), (Hoplistoderini + (Menidini + Asopinae)), and ((Sephelini + Halyini) + (Caystrini + (Cappaeini + (*Placosternum* + Phyllocephalini)))). The relationships within Phyllocephalini were (*Chalcopis* + (*Dalsira* + (*Gonopsimorpha* + *Gonopsis*))). Our results provide valuable molecular data for further phylogenetic analyses of Pentatomidae.

## Introduction

Pentatomidae is one of the largest and most diverse families in Heteroptera with respect to morphology and behavior (Rider et al., 2018). Numerous species are herbivorous, harming several important crops and causing significant economic losses around the world. For example, *Erthesina fullo* (Thunberg, 1783) is widely distributed in Asia and feeds on over 57 host plants in approximately 30 families in China (Mi et al., 2020). In addition, most species of Asopinae (Heteroptera: Pentatomidae) are predatory stink bugs that feed on the larvae of various Lepidoptera, Coleoptera, and Hemiptera, such as *Zicrona caerulea* (Linnaeus, 1758) and *Picromerus griseus* (Dallas, 1851) (Zhao Q. et al., 2017; Zhao et al., 2020).

Recent phylogenetic studies based on multigene fragments have rejected the monophyly of Pentatomidae, whereas earlier studies based on morphology and molecules supported the monophy of Pentatomidae (Grazia et al., 2008; Roca-Cusachs et al., 2021). The early classification of Pentatomidae subfamilies and tribes was mostly based on morphological data, Rider et al. (2018) divided the family into ten subfamilies and provided an extensive description and discussion of the morphology of each subfamily and tribe within Pentatomidae. However, phenotypic plasticity was a major obstacle to morphological taxonomy (Barao et al., 2013; Sheikh, 2017; Sharif et al., 2020; Genevcius et al., 2021). With the widespread application of molecular technology, molecular data are more and more used in classification. Genevcius et al. (2021) made a preliminary discussion on the main phylogenetic relationships within Pentatomidae based on a combination of morphological and molecular data. In the most detailed analysis to date, which examined the phylogenetic relationships of the whole family of Pentatomidae based on mitochondrial (*cox1* and *16S rRNA*) and nuclear gene fragments (*18S* and *28S rRNA*), Cyrtocorinae was excluded from ten subfamilies. (Roca-Cusachs et al., 2021). Nonetheless, because only *Gonopsis* Amyot & Serville, 1843 and *Minchamia* Gross, 1976 were included, the gene fragment-based analysis did not recover the phylogenetic relationships within Phyllocephalini (Pentatomidae: Phyllocephalinae) (Roca-Cusachs et al., 2021). Obviously, additional data are needed to resolve the relationships among some genera, tribes, and subfamilies of Pentatomidae.

Phyllocephalini is the largest tribe in Phyllocephalinae, with 167 species in 32 genera that feed on gramineous plants, but only 11 species in six genera have been reported in China (Kamaluddin and Ahmad, 1988; David and Zheng, 2005; Rider et al., 2018). Unlike other tribes, the tribe lacks unique and remarkable diagnostic features. Members of Phyllocephalini can be distinguished by a somewhat ovate body, short shovel-shaped head, broadly ligulate scutellum, and quadrangular pygophore; however, these characteristics may also apply to Cressonini (Linnavuori, 1982; Kamaluddin and Ahmad, 1988; Rider et al., 2018). Since the 1980s or early 1990s, systematic analyses of Phyllocephalini are rare (Linnavuori, 1982; Ahmad and Kamaluddin, 1988; Kamaluddin and Ahmad, 1988; Ahmad, 1990). The taxonomic status of the *Chalcopis glandulosus* (Wolff, 1811) has not been determined, with some Chinese scholars suggesting that it should be placed in the *Metonymia* Kirkaldy, 1909, which is a junior synonym of the *Dalsira* Amyot & Serville, 1843, while Linnavuori suggested that it should be placed in the *Chalcopis* (Kirkaldy, 1909), which is quite different from *Metonymia* in terms of morphological characteristics (Yang, 1962; David and Zheng, 2005). Accordingly, intensive sampling and genome sequencing of species belonging to Phyllocephalini are needed (Zheng et al., 2022).

Insect mitochondrial genomes (mitogenomes) are usually double-stranded circular DNA molecules, consisting of 37 genes: 13 protein-coding genes (PCGs), two ribosomal RNA genes (rRNAs), 22 transfer RNA genes (tRNAs), and a control region (CR) of variable length at the origin of replication and transcription (Wolstenholme, 1992; Boore, 1999; Stephen, 2014). Mitogenomes contain information that is crucial to molecular evolution, such as base compositional bias, codon usage, and substitution rate (Yuan et al., 2022). In addition, mitogenomes are widely used in phylogenetic studies owing to their highly conserved gene arrangement and relatively high rate of evolution (Chris et al., 2006; Zhu et al., 2017; Liu et al., 2019). Furthermore, removing the third codon of PCGs can reduce the adverse effects of heterogeneity and saturation on phylogenetic analyses (Xu et al., 2021). To date, only 50 complete or near-complete mitogenomes of Pentatomidae have been reported in GenBank, just only two of which are from Phyllocephalini. Therefore, limited sampling and molecular markers may hinder phylogenetic studies of Pentatomidae at various taxonomic levels.

In the present study, we sequenced and annotated the mitogenomes of *Gonopsis coccinea* (Walker, 1868), *Gonopsimorpha nigrosignata* (Yang, 1934), and *C. glandulosus* (Wolff, 1811), representing three genera of Phyllocephalini. The features of these mitogenomes, such as the base composition and genetic structure, were also analyzed. We mapped the secondary structures of tRNAs and rRNAs of the three mitogenomes as well as the structure of the control region. The sequence and secondary structure of the RNAs of Pentatomidae were compared and analyzed. We also calculated the evolutionary rate, codon usage, and heterogeneity of PCGs in Pentatomidae. In addition, we analyzed the genetic distances among taxa at different levels in the Pentatomidae based on the *cox1* sequence and reconstructed the phylogenetic relationships based on PCGs using BI and ML. This study not only revealed the phylogenetic positions of the three genera but also provided more mitogenome data on phylogenetic relationship of Pentatomidae.

## Materials and methods

### Sampling and species identification

The specimens of *Gonopsis coccinea*, *Gonopsimorpha nigrosignata*, and *C. glandulosus* used in this study were collected in May 2020 from Chongzuo (107°23′6.72″ E, 22°22′48.16″ N), Jingxi (106°25′21.72″ E, 23°8′30.84″ N) and Fangchenggang (108°21′24.84″ E, 21°41′40.56″ N) in Guangxi Province, China, respectively, then immediately immersed in 100% ethanol, and stored at −20°C. Species were identified by Qing Zhao based on adult morphology. Subfamily and tribe classification followed the nine-subfamily classification system (Rider et al., 2018; Roca-Cusachs et al., 2021). These three newly sequenced mitogenomes were submitted to GenBank with accession numbers ON991492, ON991493, and ON991494.

### DNA extraction and sequencing

Genomic DNA was extracted from the thoracic muscle of each specimen using the HiPure universal DNA Kitt (Jisi Huiyuan biotechnology, Nanjing, China). The mitogenomes were sequenced on the Illumina NovaSeq platform. Paired-end 150-bp (PE150) sequencing yielded approximately 5.7 Gb (*Gonopsis coccinea*), 4.8 Gb (*Gonopsimorpha nigrosignata*), and 7.3 Gb (*C. glandulosus*) data. Fastp v.0.20.0 (https://github.com/OpenGene/fastp) was used to filter the original data, and high-quality clean data were obtained by removing adaptor sequences and low-quality reads (Chen et al., 2018).

### Mitochondrial genome assembly and gene annotation

The mitogenomes were assembled using SPAdes v.3.10.1 with default parameters (Bankevich et al., 2012). The three new mitogenomes were annotated using Geneious Prime 2022.1 (Kearse et al., 2012) with a *Dalsira scabrata* Distant, 1901 obtained by BLAST in the NCBI database as a reference. The PCG boundaries were manually adjusted according to the reference sequence. To ensure the accuracy of the sequences, all PCG sequences were translated to verify amino acid sequences using Muscle as implemented in MEGA v.11.0 (Tamura et al., 2021). The locations and secondary structure of tRNA genes were confirmed using the MITOS web server (Bernt et al., 2013) with the invertebrate mitochondrial code. The rRNA gene boundaries were determined by aligning with available mitogenomes of related species. The locations of the control region were identified by the boundary of neighboring genes.

The base composition and relative synonymous codon usage (RSCU) were analyzed using MEGA v.11.0. The skew of the nucleotide composition was calculated as follows: AT skew = (A− T)/(A+ T); GC skew = (G − C)/(G + C) (Perna and Kocher, 1995). The number of non-synonymous substitutions per nonsynonymous site (K_a_) and synonymous substitutions per synonymous site (K_s_) for each PCG was calculated using DnaSP v.6.12.03 (Rozas et al., 2017), with the exclusion of stop codons. Under the Kimura 2-parameter model, genetic distances between 53 Pentatomidae species were calculated for the *cox1* gene. Tandem repeats in control regions were identified using the Tandem Repeats Finder web server (Benson, 1999). The tRNA genes were also aligned using the MUSCLE algorithm in MEGA v.11.0. The G-INS-i algorithm was selected for rRNA alignment in MAFFT v.7.490 (Katoh and Standley, 2013). Conserved sites of tRNA and rRNA were calculated using MEGA v.11.0 and annotated in their secondary structures.

### Phylogenetic analyses

Phylogenies were constructed using three newly sequenced mitogenomes and 50 species representing four subfamilies (Asopinae, Pentatominae, Phyllocephalinae, and Podopinae) of Pentatomidae, with two Scutelleridae species as outgroups (Table 1). The nucleotide sequences of each gene were extracted using Geneious Prime 2022.1. All PCGs were translated into amino acid sequences with the invertebrate mitochondrial code and aligned using MUSCLE with default parameters in MEGA v.11.0. All alignments were concatenated into a single data matrix using SequenceMatrix v.1.7.8 (Vaidya et al., 2011). To determine whether the sequences contained sufficient phylogenetic information, we tested nucleotide substitution saturation and plotted transition and transversion rates against the TN93 distances for all datasets using DAMBE v.7.3.11 (Xia, 2018). Heterogeneity in sequence divergences within the two datasets was analyzed by using AliGROOVE with the default sliding window size (Kück et al., 2014). Before reconstructing the BI trees, PartitionFinder v.2.1.1 (Lanfear et al., 2017) was used to select the optimal partitioning models, and phylogenetic analyses were conducted in MrBayes v.3.2.7 (Ronquist et al., 2012) with four independent Markov chains (three heated and one cold) run for 10,000,000 generations and sampled every 1,000 cycles. The initial 25% of trees were discarded as burn-in when the average standard deviation value was below 0.01. ML trees were reconstructed using IQ-Tree v.1.6.12 (Nguyen et al., 2015). The ModelFinder (Kalyaanamoorthy et al., 2017) implemented in the IQ-Tree was used to estimate substitution models under the Bayesian information criterion (BIC), and node support was calculated through 100,000 ultrafast bootstraps. The phylogenetic trees were constructed using two datasets: 1) all codon positions of PCGs (P123), 2) first and second codon positions of PCGs (P12). The final tree was drawn using FigTree v.1.4.4 (http://tree.bio.ed.ac.uk/software/figtree/).

**TABLE 1:** The species used in phylogenetic analyses.

Family	Subfamily	Tribe	Species	Genbank number	References
Pentatomidae			Pentatomidae sp	NC037074	Tang et al. (2014)
	Pentatominae	Antestiini	*Anaxilaus musgravei*	MW679031	Unpublished
		Sephelini	*Brachymna tenuis*	NC042802	Liu et al. (2019)
		Eysarcorini	*Carbula sinica*	NC037741	Jiang, (2017)
		Catacanthini	*Catacanthus incarnatus*	NC042804	Liu et al. (2019)
		Caystrini	*Caystrus obscurus*	NC042805	Liu et al. (2019)
		Halyini	*Dalpada cinctipes*	NC058967	Xu et al. (2021)
		Carpocorini	*Dolycoris baccarum*	NC020373	Zhang et al. (2013)
		Strachiini	*Eurydema dominulus*	NC044762	Zhao et al. (2019b)
		Strachiini	*Eurydema qinlingensis*	NC044765	Zhao et al. (2019b)
		Strachiini	*Eurydema gebleri*	NC027489	Yuan et al. (2015)
		Strachiini	*Eurydema maracandica*	NC037042	Zhao et al. (2017b)
		Strachiini	*Eurydema oleracea*	NC044764	Zhao et al. (2019b)
		Strachiini	*Eurydema liturifera*	NC044763	Zhao et al. (2019b)
		Eysarcorini	*Eysarcoris guttigerus*	NC047222	Chen et al. (2020)
		Eysarcorini	*Eysarcoris annamita*	MW852483	Li et al. (2021)
		Eysarcorini	*Eysarcoris aeneus*	MK841489	Zhao et al. (2019a)
		Eysarcorini	*Eysarcoris gibbosus*	MW846868	Li et al. (2021)
		Eysarcorini	*Eysarcoris montivagus*	MW846867	Li et al. (2021)
		Eysarcorini	*Eysarcoris ventralis*	MT165688	Li et al. (2021)
		Eysarcorini	*Eysarcoris rosaceus*	MT165687	Li et al. (2021)
		Nezarini	*Glaucias dorsalis*	NC058968	Xu et al. (2021)
		Cappaeini	*Halyomorpha halys*	NC013272	Lee et al. (2009)
		Caystrini	*Hippotiscus dorsalis*	NC058969	Xu et al. (2021)
		Hoplistoderini	*Hoplistodera incisa*	NC042799	Liu et al. (2019)
		Menidini	*Menida violacea*	NC042818	Liu et al. (2019)
		Pentatomini	*Neojurtina typica*	NC058971	Xu et al. (2021)
		Nezarini	*Nezara viridula*	NC011755	Hua et al. (2008)
		Nezarini	*Palomena viridissima*	NC050166	Unpublished
		Pentatomini	*Pentatoma rufipes*	MT861131	Zhao et al. (2021)
		Pentatomini	*Pentatoma metallifera*	NC058972	Xu et al. (2021)
		Pentatomini	*Pentatoma semiannulata*	NC053653	Unpublished
		Pentatomini	*Placosternum urus*	NC042812	Liu et al. (2019)
		Antestiini	*Plautia fimbriata*	NC042813	Liu et al. (2019)
		Antestiini	*Plautia lushanica*	NC058973	Xu et al. (2021)
		Antestiini	*Plautia crossota*	NC057080	Wang et al. (2019)
		Carpocorini	*Rubiconia intermedia*	KP207596	Yuan et al. (2015)
	Asopinae		*Arma chinensis*	NC058611	Mu et al. (2022)
			*Arma custos*	NC051562	Mu et al. (2022)
			*Cazira horvathi*	NC042817	Liu et al. (2019)
			*Dinorhynchus dybowskyi*	NC037724	Zhao et al. (2018a)
			*Eocanthecona furcellata*	MZ440302	Unpublished
			*Eocanthecona thomsoni*	NC042816	Liu et al. (2019)
			*Picromerus griseus*	NC036418	Zhao et al. (2017a)
			*Picromerus lewisi*	NC058610	Mu et al. (2022)
			*Zicrona caerulea*	NC058303	Zhao et al. (2020)
	Podopinae	Graphosomatini	*Graphosoma rubrolineatum*	NC033875	Wang et al. (2017)
		Podopini	*Scotinophara lurida*	NC042815	Liu et al. (2019)
	Phyllocephalinae	Phyllocephalini	*Gonopsis affinis*	NC036745	Chen et al. (2017)
		Phyllocephalini	*Dalsira scabrata*	NC037374	Jiang, (2017)
		Phyllocephalini	*Gonopsimorpha nigrosignata*	ON991492	This study
		Phyllocephalini	*Gonopsis coccinea*	ON991493	This study
		Phyllocephalini	*Chalcopis glandulosus*	ON991494	This study
Scutelleridae	Scutellerinae	Scutellerini	*Chrysocoris stollii*	NC051942	Unpublished
	Eurygastrinae	Eurygastrini	*Eurygaster testudinaria*	NC042808	Liu et al. (2019)

## Results

### Mitogenome structure

**FIGURE 1:**
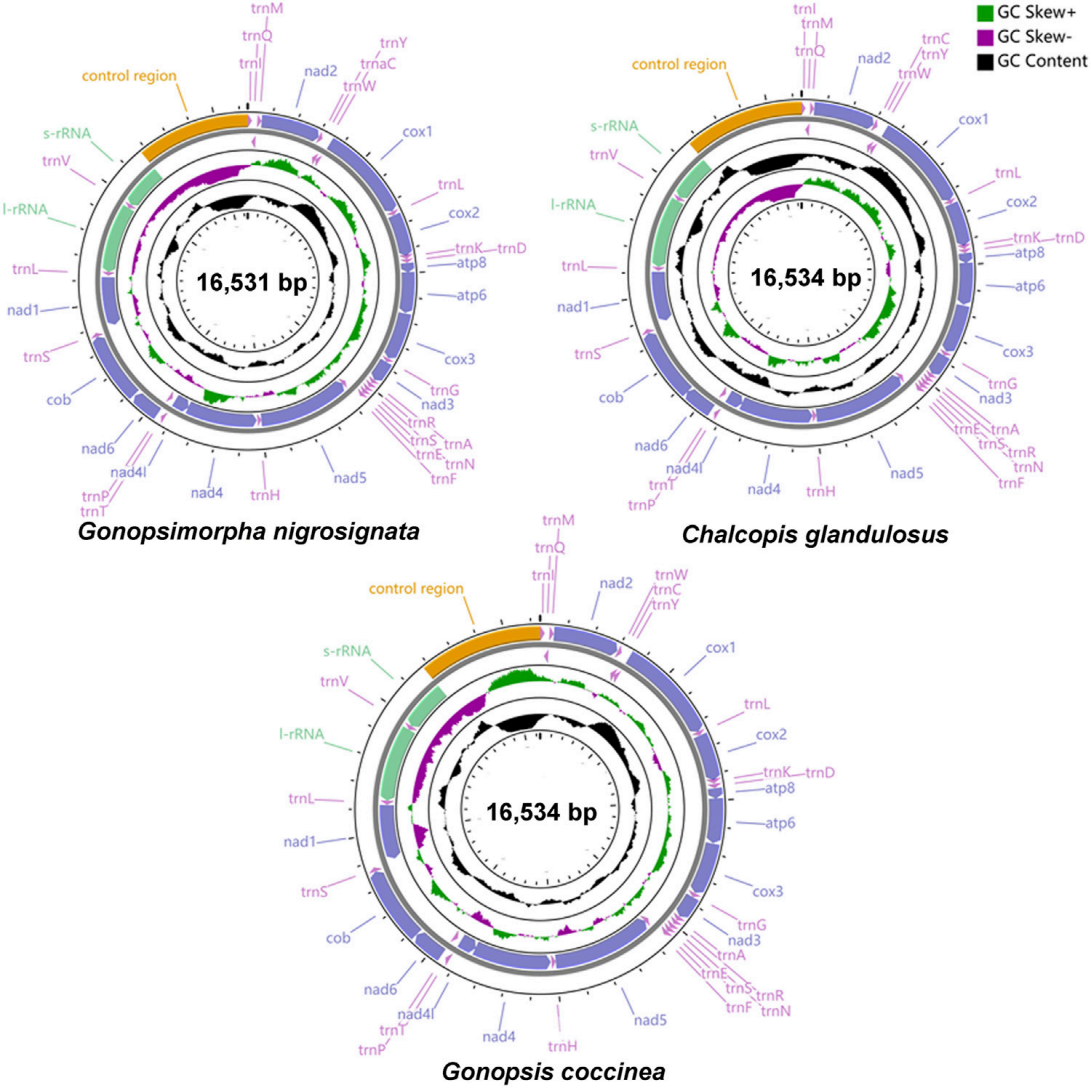
Gene structure of three complete mitochondrial genomes.

The total lengths of the mitogenomes of *Gonopsis coccinea*, *Gonopsimorpha nigrosignata*, and *C. glandulosus* were 16,534 bp, 16,531 bp, and 16,534 bp, respectively (Figure 1). The mitochondrial genomes of all three species were closed circular double-stranded molecules composed of 37 coding genes (13 PCGs, two rRNAs, and 22 tRNAs) and a non-coding control region (Figure 1, Supplementary Table S1). The gene arrangements in the three mitogenomes were identical, with 23 genes (nine PCGs and 14 tRNAs) located on the majority strand (J-strand) and 14 genes (four PCGs, eight tRNAs and two rRNAs) on the minority strand (N-strand) (Supplementary Table S1). The nucleotide composition of whole mitogenomes showed a significant bias toward A and T bases, with A + T contents of 78.93% (*Gonopsis coccinea*), 77.13% (*Gonopsimorpha nigrosignata*), and 78.94% (*C. glandulosus*). The AT skew was positive in all cases, while the GC skew was negative (Supplementary Table S2).

The longest intergenic spacers were detected between *nad1* and *trnS1*, ranging from 22 to 28 bp in length. There was a conserved 2 bp gene spacer between *nad4L* and *trnT*, and a conserved 1 bp interval between *trnH* and *nad5*. The longest gene overlap was located between *trnC* and *trnW*, with a length of 8 bp. Several regions of conserved gene overlap were also observed in the three mitogenomes, including *atp8*/*atp6* (7 bp), *trnN*/*trnS1* (1 bp), *trnE*/*trnS1* (1 bp), and *nad4*/*nad4L* (7 bp) (Supplementary Table S1).

### Protein-coding genes

The total lengths of the 13 PCGs of *Gonopsis coccinea*, *Gonopsimorpha nigrosignata*, and *C. glandulosus* were 11,040 bp, 10,992 bp, and 11,022 bp. The nucleotide composition of the 13 PCGs showed high A+ T contents, i.e., 77.73% (*Gonopsis coccinea*), 75.81% (*Gonopsimorpha nigrosignata*), and 77.90% (*C. glandulosus*). A moderate negative AT skew and negligible positive GC skew was observed in 13 PCGs in the three mitogenomes (except in *Gonopsimorpha nigrosignata*) (Supplementary Table S2). Of all 13 PCGs in the three mitogenomes, nine (*nad2*, *cox1*, *cox2*, *atp8*, *atp6*, *cox3*, *nad3*, *nad6*, and *cob*) were located on the J-strand, and the other four (*nad5*, *nad4*, *nad4L*, and *nad1*) were found on the N-strand. Among the 13 PCGs, *atp8* (159–162 bp) was the shortest, while *nad5* (1708–1717 bp) was the longest. Most PCGs were initiated by ATN (ATT/ATA/ATG/ATC) as the start codon, while TTG was the second most common start codon, detected in *cox1*, *nad6*, (*C. glandulosus*), and *atp8*. Most PCGs ended with the complete termination codon TTA or TAG, except for *cox1*, (except in *Gonopsimorpha nigrosignata*), *cox2*, and *nad5*, which ended with the incomplete stop codon T (Supplementary Table S1).

**FIGURE 2:**
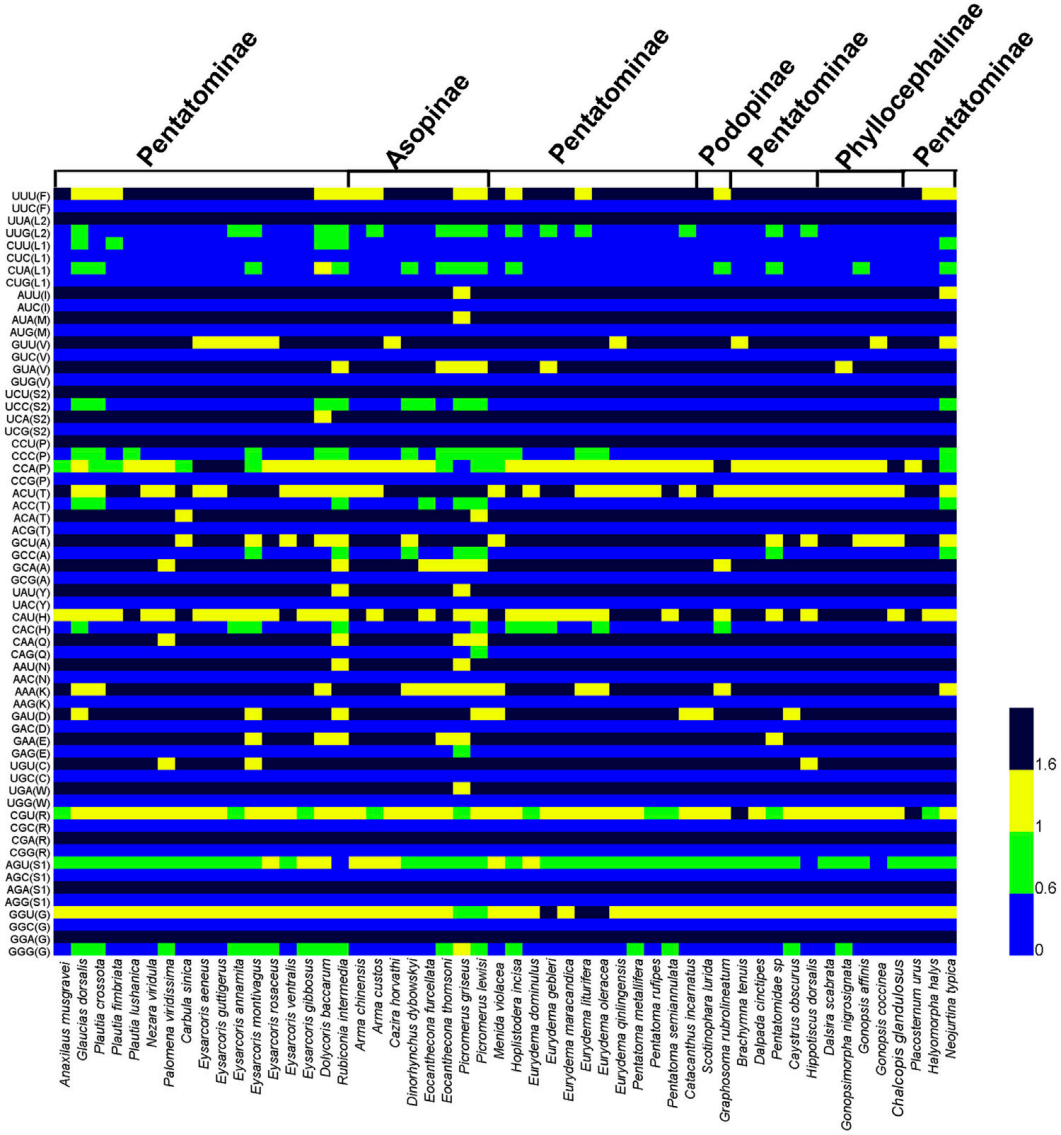
Heatmap analysis of relative synonymous codon usage (RSCU) values among the 53 species of Pentatomidae.

We calculated RSCU of PCGs for three species of Phyllocephalini, four subfamilies of Pentatomidae, and 53 species of Pentatomidae respectively (Figure 2; Supplementary Figure S1, S2). The similar RSCU pattern was observed in all the pentatomidae. Among all the codons, UUA (L2) was found to be the most frequently used codon, and we observed that most of the codons with high frequency ended in AT, indicating that a preference for AT over GC in the codon composition of Pentatomidae mitogenomes. Among the four subfamilies of Pentatomidae, the predatory Asopinae showed a weaker preference for RSCU than the other three subfamilies (Figure 2; Supplementary Figure S1).

**FIGURE 3:**
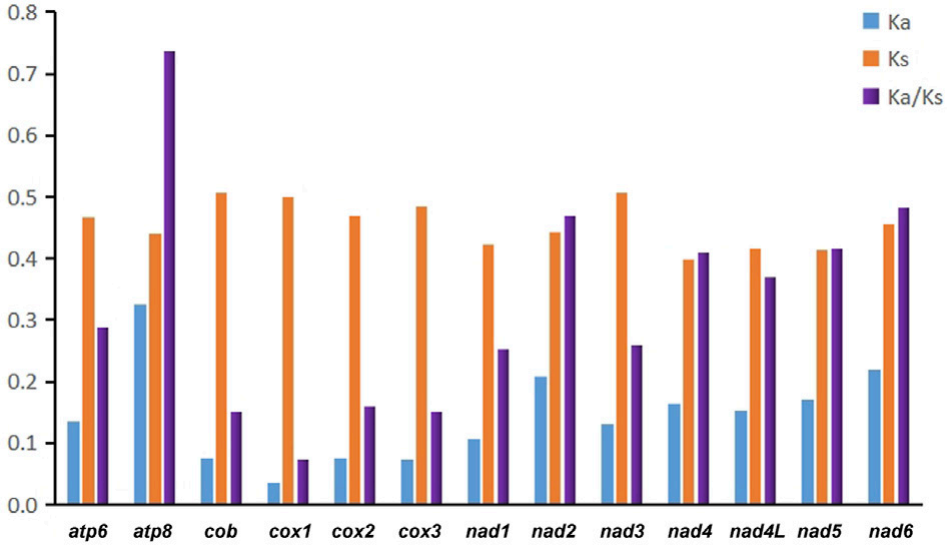
The K_a_ (non-synonymous substitutions), K_s_ (synonymous substitutions), and K_a_/K_s_ values of each PCGs in Pentatomidae.

In addition, we evaluated the synonymous substitution rate (K_s_), non-synonymous substitution rate (K_a_), and the K_a_/K_s_ ratio of each PCG to explore patterns of evolution in Pentatomidae (Figure 3). The K_a_/K_s_ ratios for all 13 PCGs were below 0.74, indicating that the genes were under purifying selection. Therefore, all of the PCGs could be used to analyze phylogenetic relationships within Pentatomidae. Among the PCGs, *atp8* had the highest K_a_/K_s_ ratio, while *cox1* had the lowest value. Owing to its slow rate of evolution, *cox1* is commonly used for barcoding analyses and used for genus or species identification (Hebert et al., 2004). We also calculated pairwise genetic distances at different taxonomic levels in Pentatomidae based on *cox1* sequences and compared the results with those for Pentatomomorpha and Heteroptera (Park et al., 2011; Tembe et al., 2014) (Table 2). We observed a hierarchical increase in the mean genetic distance across different taxonomic levels. The mean interspecific genetic distance was 15.86% with a range of 2.27%–22.05%. The genetic distance amongst genera averaged 16.30%, with a range of 11.03%–21.02%. The average genetic distance between subfamilies was 16.80%, with a range of 16.06%–17.66%.

**TABLE 2:** The genetic distances (K2P) at various taxonomic levels in Pentatomidae.

	Range (%)	Mean dist (%)
Species	2.27–22.05	15.86
Genera	11.03–21.02	16.30
Subfamilies	16.06–17.66	16.80

### Transfer and ribosomal RNAs

**FIGURE 4:**
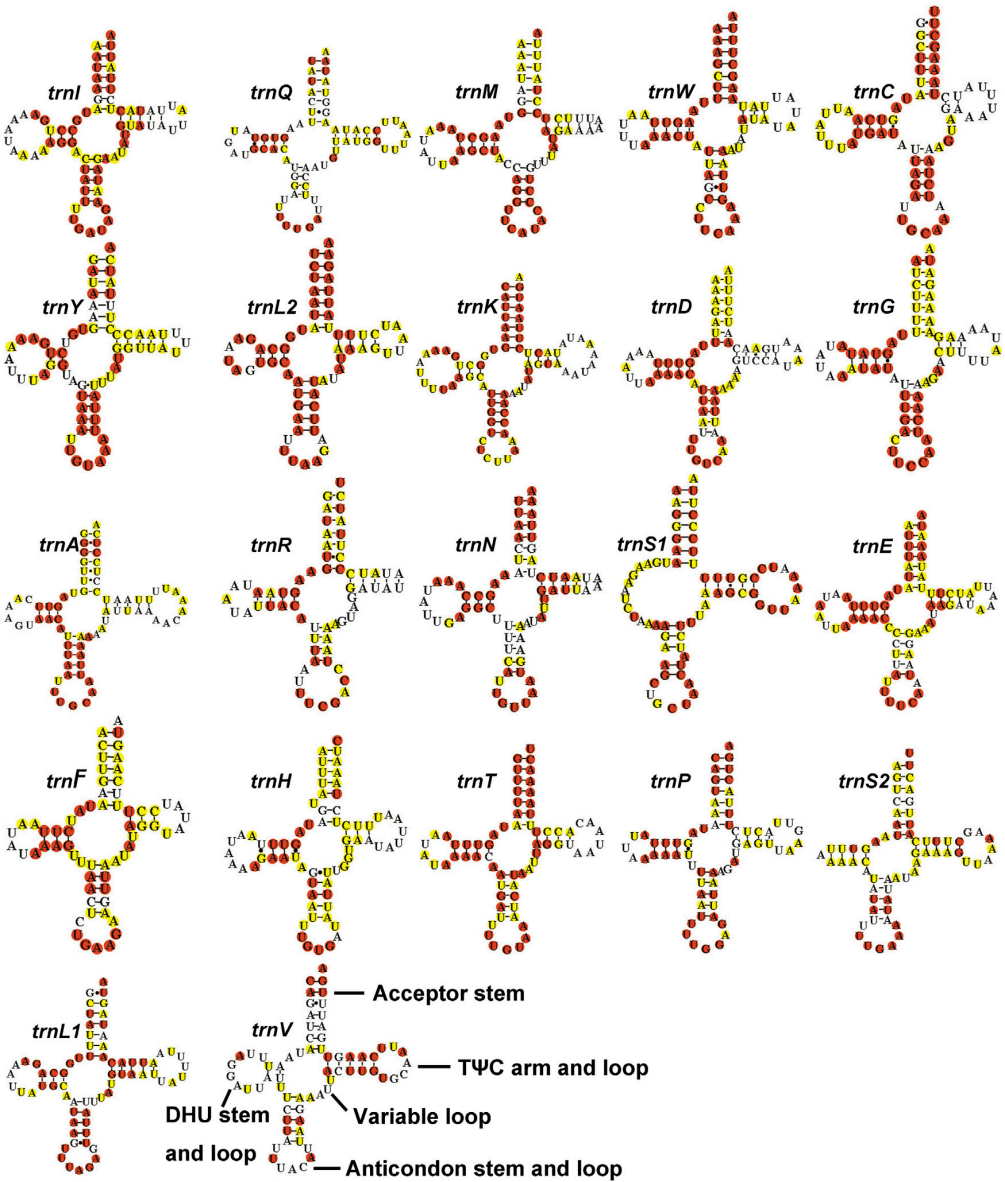
The potential secondary structure of the tRNA in *Gonopsis coccinea*. Conserved sites are marked red in Pentatomidae and yellow in Phyllocephalinae.

The full lengths of 22 tRNAs of *Gonopsis coccinea*, *Gonopsimorpha nigrosignata*, and *C. glandulosus* were 1,477 bp, 1,478 bp, and 1,473 bp, respectively. The 22 tRNAs showed high A+ T contents of 78.27% (*Gonopsis coccinea*), 76.59% (*Gonopsimorpha nigrosignata*), and 78.28% (*C. glandulosus*), and positive AT and GC skews were also observed (Supplementary Table S2). Fourteen tRNAs (*trnI*, *trnM*, *trnW*, *trnL2*, *trnK*, *trnD*, *trnG*, *trnA*, *trnR*, *trnN*, *trnS1*, *trnE*, *trnT*, and *trnS2*) were encoded by the J-strand and the remaining eight (*trnQ*, *trnC*, *trnY*, *trnF*, *trnH*, *trnP*, *trnL1*, and *trnV*) were encoded by the N-strand. The lengths of the 22 tRNAs of the three mitogenomes ranged from 63 to 74 bp (Supplementary Table S1). All tRNAs could be folded into typical cloverleaf structures containing of four stems, except for *trnS1*, whose dihydropyridine (DHU) stem was replaced with a simple loop (Figure 4). In the secondary structures of tRNAs of the Pentatomidae, an anticodon loop (7 nt), anticodon stem (5 bp, except for *trnS1*), and acceptor stem (7 bp) were conserved in length, while the lengths of the DHU stem (2–4 bp) and TψC stem (3–5 bp, except for *trnC*) were variable. Among the 22 tRNAs in the Pentatomidae, *trnL2* was the most conserved, with the complete conservation of four stems and only a small number of unconserved bases in the loop, while *trnV* was the least conserved. All tRNAs of the Pentatomidae use the standard anticodon, except for *trnV* of *Gonopsis affinis* (Uhler, 1860). Additionally, we observed mismatched base pairs (GT), which play an important role in maintaining the stability of the tRNA secondary structure and can be restored by post-transcriptional editing (Ojala et al., 1981).

**FIGURE 5:**
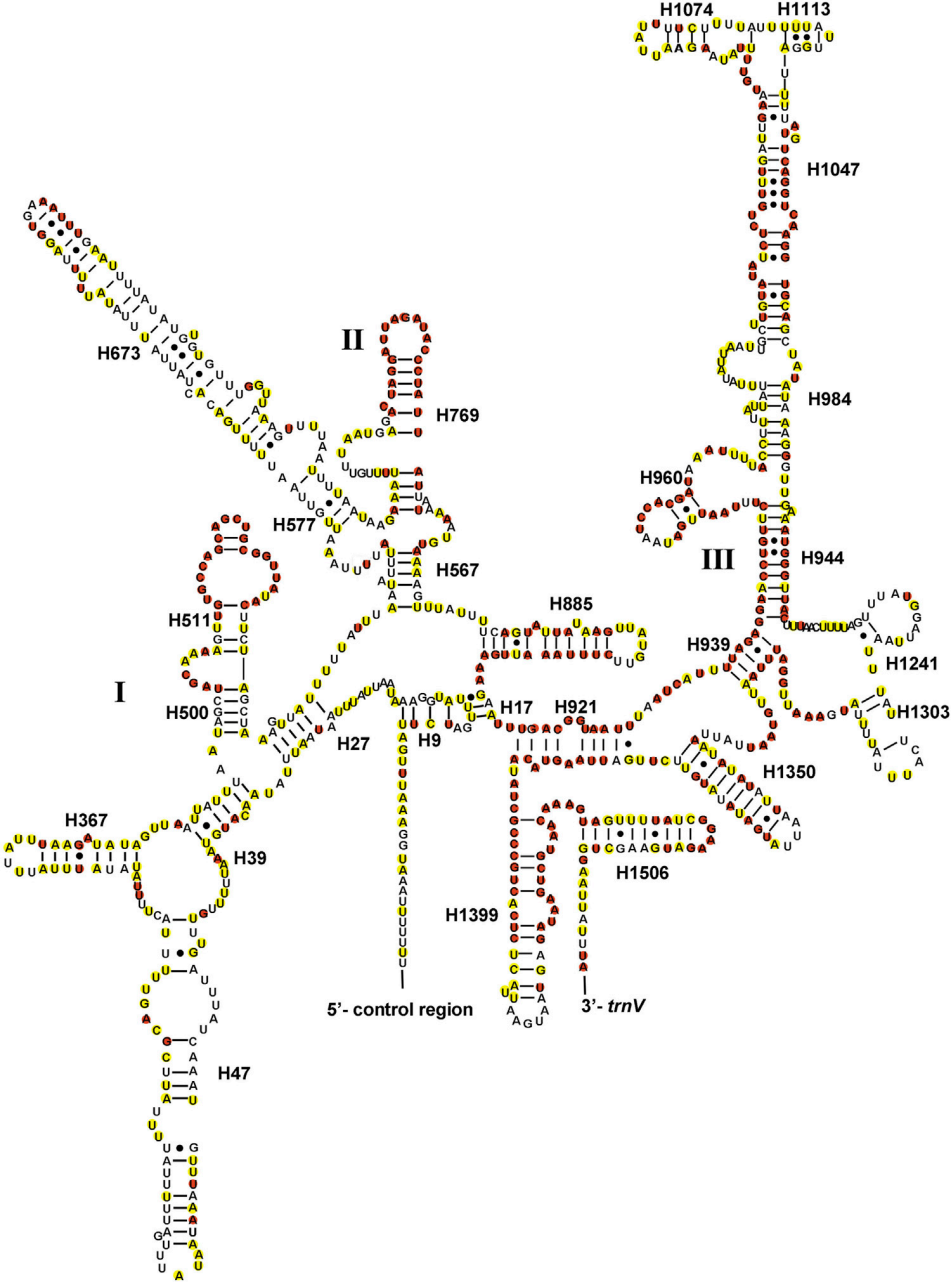
The potential secondary structure of the *12S rRNA* in *Gonopsis coccinea*. Conserved sites are marked red in Pentatomidae and yellow in Phyllocephalinae.

**FIGURE 6:**
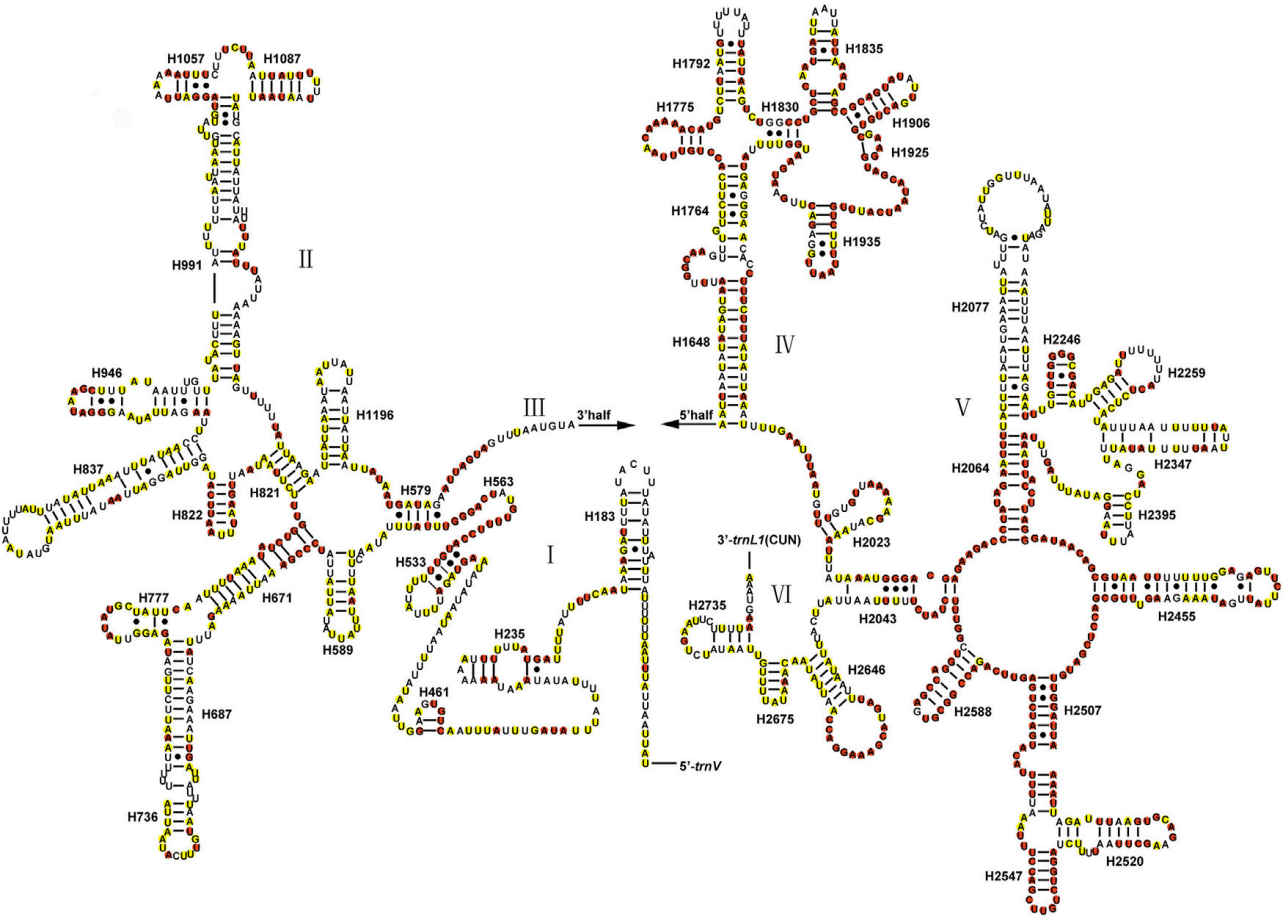
The potential secondary structure of the *16S rRNA* in *Gonopsis coccinea*. Conserved sites are marked red in Pentatomidae and yellow in Phyllocephalinae.

The total lengths of two rRNAs of *Gonopsis coccinea*, *Gonopsimorpha nigrosignata*, and *C. glandulosus* were 2,104 bp, 2,079 bp, and 2,080 bp. The two rRNAs showed high A+ T contents of 79.42% (*Gonopsis coccinea*), 78.69% (*Gonopsimorpha nigrosignata*), and 80.14% (*C. glandulosus*), and a negative AT skew and positive GC skew were also observed (Supplementary Table S2). The two rRNAs were encoded on the N-strand in the three mitogenomes. The *12S rRNA* gene ranged from 797 bp (*Gonopsimorpha nigrosignata*) to 815 bp (*Gonopsis coccinea*) in length and was located between *trnV* and the control region (Supplementary Table S1). In the *12S rRNA* secondary structure of Pentatomidae, domain III was more conserved than domains I and II (Figure 5). The *16S rRNA* gene was observed between *trnL1* and *trnV*, with lengths ranging from 1,282 bp to 1,289 bp. In the secondary structure of *16S rRNA*, domains IV and V were more conserved than domains I, II, and VI within Pentatomidae (Figure 6).

### Control region

**FIGURE 7:**
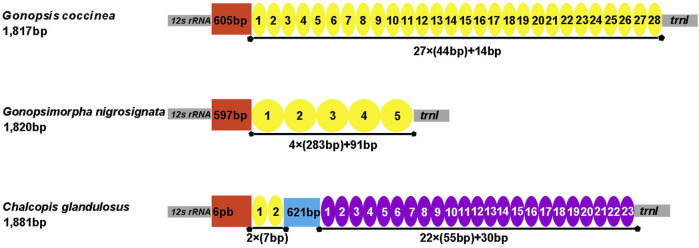
Organization of the control region in the three mitochondrial genomes. The location and copy number of tandem repeats are shown by a colored oval with repeat length. Non-repeat regions are shown by the colored box with sequence size inside.

The control region is the largest non-coding region. The control regions of the three mitogenomes were located between *12S rRNA* and *trnI*, with lengths of 1,817 bp (*Gonopsis coccinea*), 1,820 bp (*Gonopsimorpha nigrosignata*), and 1,881 bp (*C. glandulosus*), respectively. All three species showed extremely high A+ T contents of 85.25% (*Gonopsis coccinea*), 82.53% (*Gonopsimorpha nigrosignata*), and 83.63% (*C. glandulosus*). *Gonopsis coccinea* showed moderate positive AT and negative GC skews, while the remaining species showed a slight negative AT skew and strong negative GC skew. Only one type of tandem repeat unit was found in the control region of *Gonopsis coccinea* and *Gonopsimorpha nigrosignata*, with lengths of 44 bp and 283 bp, respectively. Two types of tandem repeat units were observed in the control region of *C. glandulosus*, separated by a 621 bp non-repeat region (Figure 7).

### Phylogeny

#### Tests of substitution saturation and heterogeneity in sequence divergence

**FIGURE 8:**
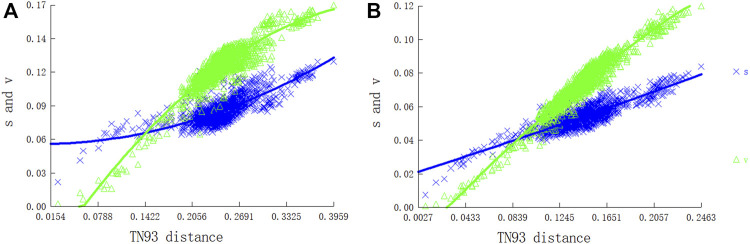
Nucleotide substitution saturation plots of all mitochondrial protein-coding genes: **(A)** All codon positions; **(B)** First and second codon positions.

We evaluated substitution saturation in the two PCG datasets (P12 and P123) before the phylogenetic analysis. The saturation index *I*_SS_ (developed by Xia) for the two datasets was significantly lower than those for a symmetric (*I*_ss.cSym_) and asymmetric (*I*_ss.cAsym_) topology, indicating that the two data sets were not saturated. The *I*_SS_ value of P123 was higher than that of P12, indicating that the P123 dataset was more saturated than P12. Furthermore, there was a linear correlation between the base transition and transversion rates and the modified genetic distance, which indicated that the nucleotide sequences of PCGs were generally unsaturated and suitable for phylogenetic analyses of Pentatomidae (Figure 8).

**FIGURE 9:**
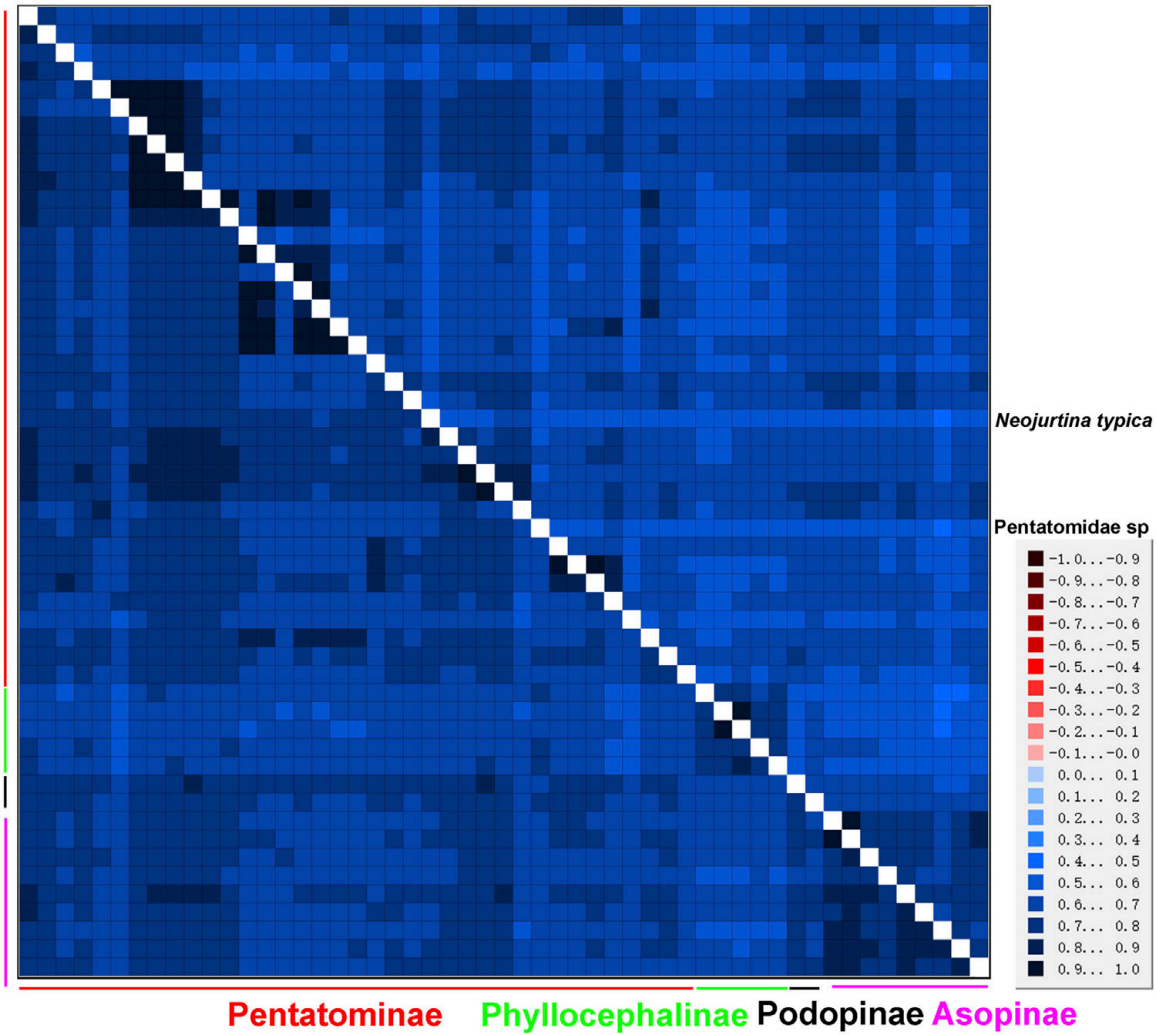
AliGROOVE analysis of 53 Pentatomidae species based on PCG12 (lower triangle) and PCG123 (upper triangle). The mean similarity score between sequences is represented by colored squares, based on AliGROOVE scores ranging from −1, which indicates a great difference in rates from the remainder of the data set, that is, heterogeneity (red coloring), to +1, which indicates rates that matched all other comparisons (blue coloring).

An AliGROOVE analysis of the two datasets showed that the degree of heterogeneity of the P123 dataset was higher than that of the P12 dataset. The third codon position had higher heterogeneity, as expected. Among the four subfamilies of Pentatomidae, Phyllocephalinae showed the highest heterogeneity. *Neojurtina typica* Distant, 1921 showed high heterogeneity within Pentatominae (Figure 9).

#### Phylogenetic analyses

**FIGURE 10:**
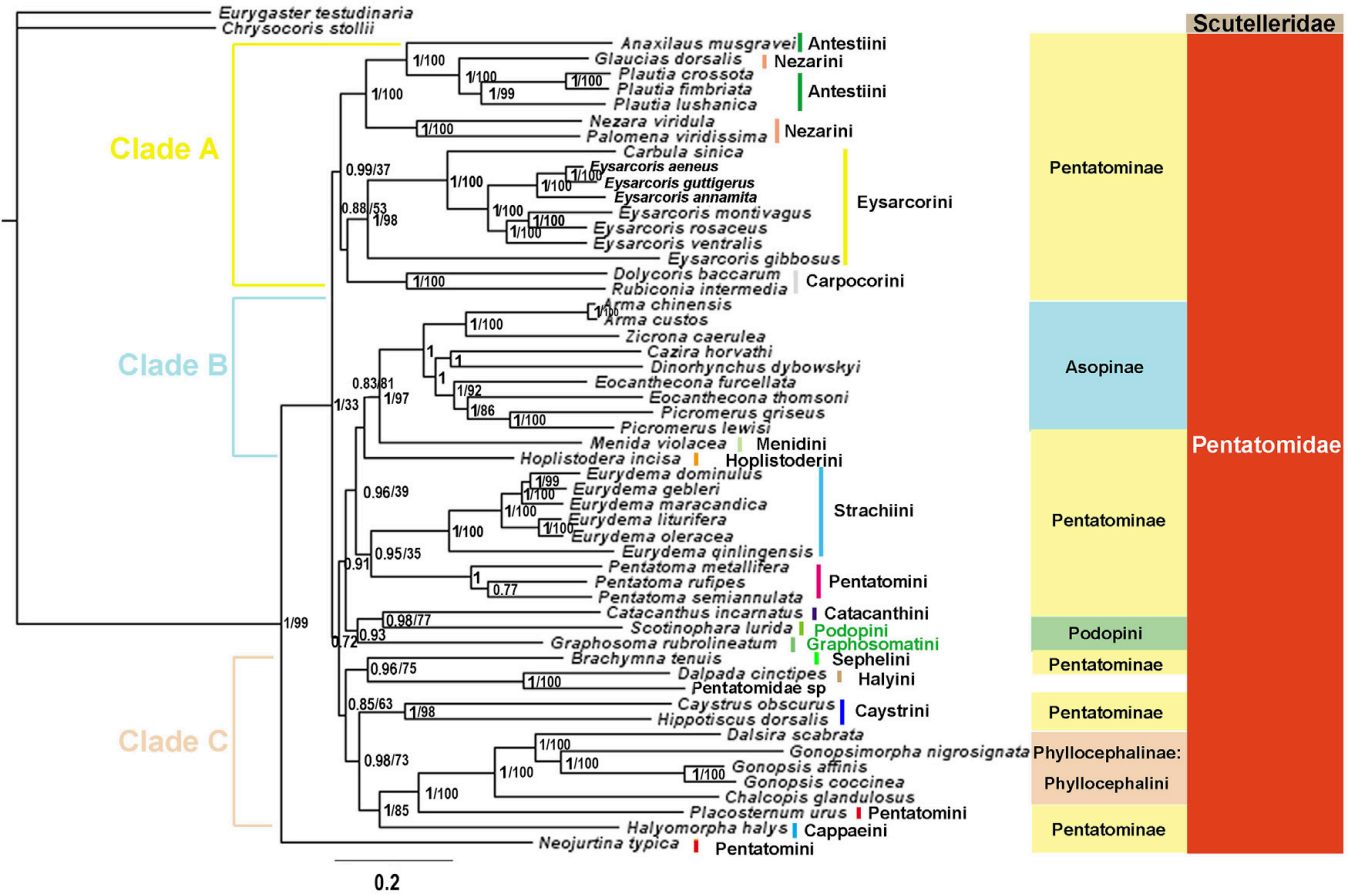
The phylogenetic trees of tribes within Pentatomidae were constructed from DNA sequences of 13 PCGs using BI and ML methods. Numbers on branches are posterior probabilities (PP, left) and bootstrap (BS, right). Note: Branches without BS values indicate that the topology is only supported by BI.

**FIGURE 11:**
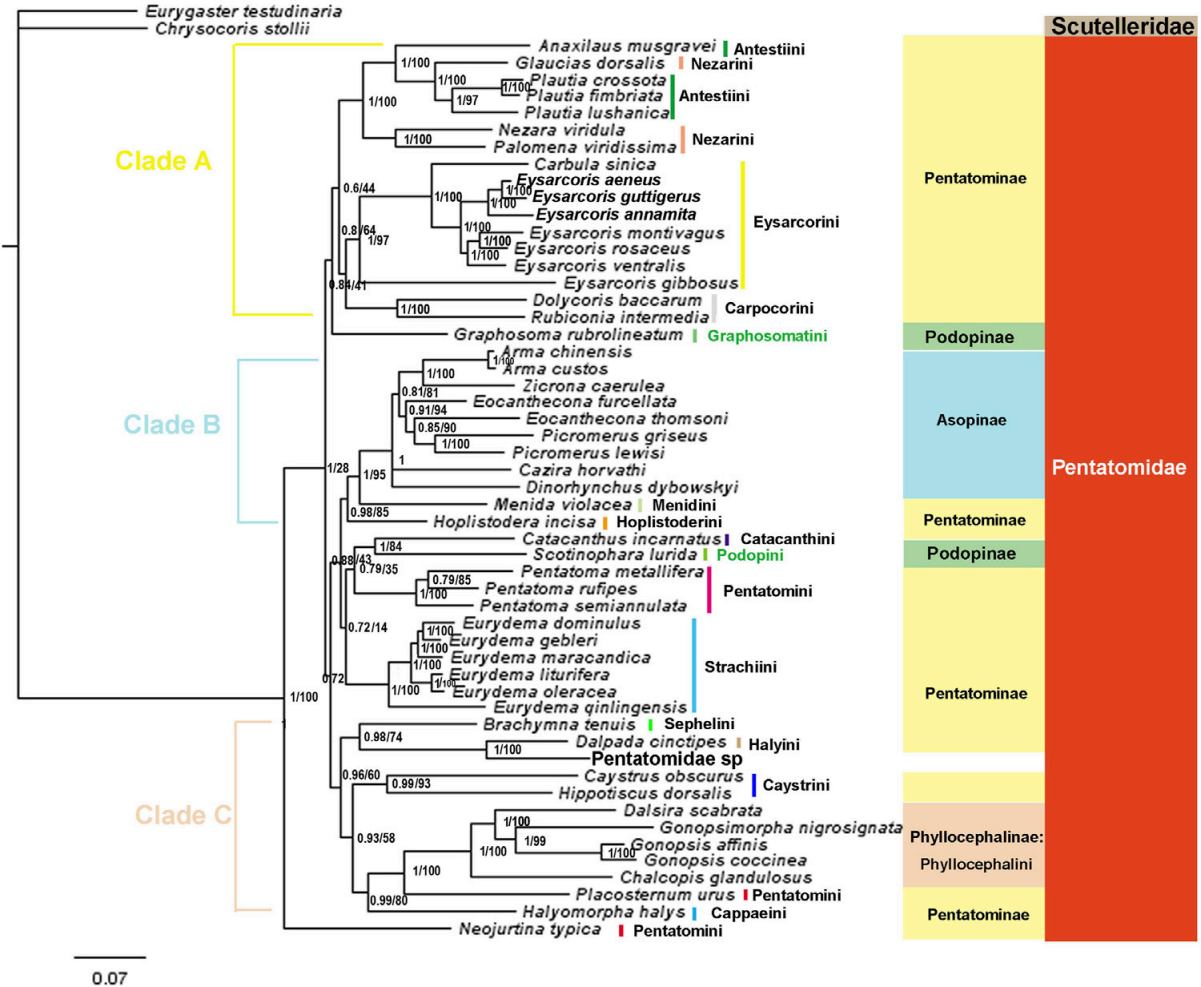
The phylogenetic trees of tribes within Pentatomidae were constructed from DNA sequences of 13 PCGs excluding the third site using BI and ML methods. Numbers on branches are posterior probabilities (PP, left) and bootstrap (BS, right). Note: Branches without BS values indicate that the topology is only supported by BI.

We constructed phylogenetic trees of Pentatomidae based on the two data sets using BI and ML (Figure 10, 11, Supplementary Figure S3, S4). The topological structure of the four trees was highly consistent, and most clades had high posterior probabilities. All analyses showed that *N. typica* was the earliest diverging lineage within Pentatomidae. Asopinae and Phyllocephalinae were recovered as monophyletic groups with strong support values and high internal node support values. Asopinae cannot be subdivided into tribes, and the topology of the internal genera in the P123 tree was ((*Arma* + *Zicrona*) + ((*Cazira* + *Dinorhynchus*) + (*Eocanthecona* + *Picromerus*))). However, the sister group relationship of *Cazira* and *Dinorhynchus* was not recovered in the remaining three phylogenetic trees. The species used for the phylogenetic analysis of the Phyllocephalinae all belonged to Phyllocephalini. The internal topology of Phyllocephalini was consistently (*Chalcopis* + (*Dalsira* + (*Gonopsimorpha* + *Gonopsis*))) with high internal node support. The monophyly of both Pentatominae and Podopinae was rejected, and tribal-level relationships were partially recovered in our analyses. Eysarcorini and Strachiini were recovered as monophyletic with strong support, while the monophyly of Antestiini, Nezarini, and Pentatomini was rejected. The monophyly of the remaining tribes was not clear owing to the limited availability of mitogenomes in the NCBI database.

## Discussion

### Mitogenome organization and characteristics

Most PCGs in the three mitogenomes used the common triplet codon ATN as the start codon, while *cox1* and *atp8* used TTG as the start codon, which was also found in the mitochondrial genomes of many species within Pentatomidae (Zhao W. et al., 2017; Wang et al., 2017). In addition, *nad6* of *C. glandulosus* used TTG as the start codon, whereas ATN was used in the other two species. Most PCGs utilized TAA as the stop codon, while three PCGs (*cox1*, *cox2,* and *nad5*) used a single T, as observed in most Pentatomidae mitogenomes (Zhao W. et al., 2017). Nevertheless, *nad1*, *nad4*, and *cob* of *C. glandulosus* used TAG as the stop codon. Our results suggest that some PCGs in *C. glandulosus* exhibit distinct start and stop codons from homologues in the other two species of Phyllocephalini. In addition, the genetic distances calculated based on the *cox1* gene showed that, among the four subfamilies, Asopinae and Phyllocephalinae were the most distantly related, as reflected in the rostrum structures. However, Pentatominae and Podopinae were closely related. This may be one reason why the species of Podopinae in the phylogenetic tree were mixed in Pentatominae. *Glaucias* Kirkaldy, 1908 and *Picromerus* were the most distantly related genera, while *Arma* Hahn, 1832 and *Zicrona* was the most closely related. Among species, the genetic distance between *Arma custos* (Fabricius, 1794) and *Arma chinensis* Fallou, 1881 was the smallest, they have been synonymized (Zhao et al., 2018b).

Most tRNAs of the Pentatomidae have a typical cloverleaf secondary structure, except for the lack of the DHU stem in *trnS1*, which has also been reported in previous studies of Pentatomidae insects (Zhao et al., 2018a; Mu et al., 2022). The stem region is more conserved than the loop region, suggesting a difference in selective pressure between the two regions. The stem region sequence may be under slower selection to maintain its secondary structure, while the loop region evolves more rapidly (Zhang and Ryder, 1993). The secondary structures of the two rRNAs are conserved within Pentatomidae, and sequence conservation of *16S rRNA* (37.75%) was higher than that of *12S rRNA* (28.42%). H47 and H673 have a high degree of variation in sequence and secondary structure in *12S rRNA* of Pentatomidae. In particular, H47 shows a highly variable structure, suggesting that it shows taxonomic specificity and can be used for classification within Pentatomidae (Yuan et al., 2015). The H921–960, H1047, and H1399 helices at the 3′ end of *12S rRNA* were conserved within Pentatomidae. The H2077 helix of *16S rRNA* is highly variable in terms of its sequence and secondary structure within Pentatomidae, while six helices (H563, H1775, H2064, H2507, H2547, and H2588) were conserved. Previous studies have shown that the variable length and differentiated tandem repeat units in the control region provide a basis for use as molecular markers to study the evolution and population genetics of pentatomoid species (Wang et al., 2015; Yuan et al., 2015). The control regions of *Gonopsis coccinea* and *Gonopsimorpha nigrosignata* were similar in length and each had only one type of tandem repeat unit. The control region of *C. glandulosus* was much longer than those of the other two species, and two types of repeat units were observed.

### Phylogenetic relationships among Pentatomidae

The phylogenetic trees based on two datasets using BI and ML methods were completely consistent except for the position instability of *Graphosoma rubrolineatum* (Westwood, 1837), and were completely consistent with those obtained from PCGs and rRNAs (P123RNA) by BI and ML method (Li et al., 2021). In our study, the third codon position of PCG gene showed higher saturation and heterogeneity compared with the first and second codon positions, which may adversely affect the phylogenetic results. Removing the third site and Bayesian analysis under mixed heterogeneous model is a good solution (Yuan et al., 2015; Xu et al., 2021). However, removing the third site of PCG decreased the nodal support of phylogenetic trees. In the previous heterogeneity analysis of Pentatomoidea, Pentatomidae showed the lowest heterogeneity, and our phylogenetic results under the homogeneous model were completely consistent with those under the heterogeneous model (Liu et al., 2019; Xu et al., 2021). This suggests that the third site of PCGs contains information critical to solving the phylogenetic relationships of the Pentatomidae, and that the Bayesian approach under both heterogeneous and homogeneous models is equally expressive for this group (Zhao W. et al., 2019). In our phylogenetic tree, *Neojurtina* was the first lineage to diverge and formed a sister group with high node support with the remaining species of Pentatomidae, which was also observed in the phylogenetic tree of Pentatomoidea constructed based on P123RNA and P12RNA datasets under heterogeneous mixed model (Xu et al., 2021).

All phylogenetic analyses strongly supported the following relationship within Phyllocephalini: (*Chalcopis* + (*Dalsira* + (*Gonopsimorpha* + *Gonopsis*))). These results, combined with the structure of the control region and the initiation and termination codons, suggest that *Gonopsimorpha* and *Gonopsis* are more closely related to each other than to *Chalcopis*. *C. glandulosus* was the earliest separate clade within Phyllocephalini and did not form a sister group with *Dalsira* (junior synonym of the *Metonymia*), which supported morphologic idea that it belongs to *Chalcopis* (David and Zheng, 2005)*.* Phyllocephalini did not form an independent clade. Phyllocephalini formed a sister group with *Placosternum* Amyot & Serville, 1843 with high support and then converged with *Halyomorpha* (Mayr, 1864). The closer relationship between the Phyllocephalini and *Halyomorpha* was also supported by BI and ML methods based on P123, P12, P12RNA and P123RNA datasets (Chen et al., 2017; Zhao et al., 2020; Zhao et al., 2021).

The species assigned to clade A all belonged to Pentatominae and its topological structure was (((*Anaxilaus* + (*Plautia* + *Glaucias*)) + (*Nezara* + *Palomena*)) + (Eysarcorini + Carpocorini)). Neither Antestiini nor Nezarini formed a monophyletic group; however, they were closely related. Rider temporarily placed *Plautia* (Stål, 1865) in Antestiini, and our phylogenetic results supported this morphology-based view that *Plautia* may bridge the gap between Antestiini and the Nezarini (Rider et al., 2018). *E. gibbosus* (Jakovlev, 1904) is the first diverging clade within Eysarcorini, which did not cluster with other species of *Eysarcoris* Hahn, 1834. This result is consistent with previous results based on molecular and morphological evidence; accordingly, we support the previous proposal that *E. gibbosus* should be transferred to *Stagonomus* Gorski, 1852 (Roca-Cusachs and Jung, 2019; Li et al., 2021). In this study, *Dolycoris baccarum* (Linnaeus, 1758) formed a sister group with *Rubiconia intermedia* (Wolff, 1811)*,* which has previously been placed in Eysarcorini, supporting the most recent morphological attribution of them to Carpocorini (Puton, 1869; Rider et al., 2018). Eysarcorini and Carpocorini are similar with respect to morphological characteristics and were sister groups in our phylogenetic results, indicating that the species in the two tribes are closely related (Rider et al., 2018; Li et al., 2021).

The topology of clade B was (Hoplistoderini + (Menidini + Asopinae)). In this study, the relationships among the genera in the Asopinae obtained by using the mixed homogeneity model are completely consistent with the results of the mixed heterogeneity model (Xu et al., 2021). The sister group relationship between Menidini and Asopinae has also been found in phylogenetic analyses based on nuclear and mitochondrial genes, consistent with the previously proposed hypothesis, and there were similar pseudoclaspers structures between the two groups (Gapud, 1981; Roca-Cusachs et al., 2021). The topology of clade C was ((Sephelini + Halyini) + (Caystrini + (Cappaeini + (*Placosternum* + Phyllocephalini)))). This was consistent with the previous phylogenetic relationships of Pentatomomorpha under the site-heterogeneous mixture model and Pentatominae under the homogenous model based on the sequences of the 13 PCGs and two rRNAs (Liu et al., 2019; Li et al., 2021). In this study, the three genera (*Pentatoma*, *Placosternum*, *Neojurtina*) of Pentatomini did not converge, and only the three species of *Pentatoma* converged. The sternal structure of the Old World genus *Placosternum* is somewhat more reminiscent of the New World South American genus *Evoplitus*, and sternal structure of genus *Evoplitus* strikingly similar to the Old World tribe Rhynchocorini (Rider et al., 2018). *Neojurtina* was only temporarily placed in Pentatomini, while *Pentatoma* has always been well placed in Pentatomini (Rider et al., 2018). Therefore, more evidence is needed to determine the phylogenetic position of *Placosternum* and *Neojurtina* in Pentatomidae.

Owing to the small number of mitogenomes available, the phylogenetic relationships of Podopinae are unresolved, and stable clustering was not obtained in the four phylogenetic trees. In the phylogenetic BI tree based on the P123 data set, *Graphosoma* was clustered with *Scotinophara*, which was a sister group of clade A in the remaining three trees. In terms of morphology, *G. rubrolineatum* belongs to the Podopinae (Yang, 1962; Rider et al., 2018). In the previous studies based on mitochondrial genome, most of them selected only *G. rubrolineatum* as the representative species of the Podopinae, so the Podopinae is mixed in the Pentatominae (Chen et al., 2017; Mu et al., 2022; Yuan et al., 2015; Zhao et al., 2021; Zhao et al., 2020; Zhao Q. et al., 2019; Wang et al., 2017; Wang et al., 2019). However, in the study based on the P123RNA and P12RNA datasets, the two representative species of the Podopinae were selected, and the *G. rubrolineatum* did not converge with other species of the Podopinae (Xu et al., 2021). These results combined with our phylogenetic results indicate that the reason for the lack of stable position of *G. rubrolineatum* lies in the limited mitogenome and the limitation of molecular marker selection. We think the way to solve this problem is to sequence more mitochondrial genomes of the Podopinae and more molecular markers such as nuclear genes. Although the position of *G. rubrolineatum* differed between the four phylogenetic trees, *Scotinophara lurida* (Burmeister, 1834) and *Catacanthus incarnatus* (Drury, 1773) were sister groups in all trees, which are also observed in the molecular clock analysis (Liu et al., 2019). In addition, the phylogenetic relationship of *Eurydema* Laporte, 1833 was completely consistent with the results of morphological studies (Zhao W. et al., 2019).

Since the 1990s, few phylogenetic studies have focused on Phyllocephalini. Owing to the limited number of mitogenomes, the internal phylogenetic relationships are not clear. Earlier studies based on morphological characteristics are expected to pay more attention to the molecular phylogenetic study of this group (Rider et al., 2018). In our study, three mitogenomes from Phyllocephalini were sequenced, representing a substantial addition to the two existing mitogenomes of Phyllocephalini. In addition, our results clarified partially the phylogenetic relationships within Pentatomidae at the subfamily, tribe, and genus levels. It is necessary to sequence more mitochondrial genomes, including the other subfamilies and genus groups, to better understand the molecular evolution and phylogenetic relationships of this group.

## Data Availability

The datasets presented in this study can be found in online repositories. The names of the repository/repositories and accession number(s) can be found in the article/Supplementary Material.
