# Supplementary material for: Comparative analysis and phylogeny of mitochondrial genomes of Pentatomidae (Hemiptera: Pentatomoidea)
Source: Front Genet. 2022 Nov 11;13:1045193. doi: 10.3389/fgene.2022.1045193 (PMC9692006; doi:10.3389/fgene.2022.1045193)
Supplement: Supplementary file 1 [file Presentation1.pdf]

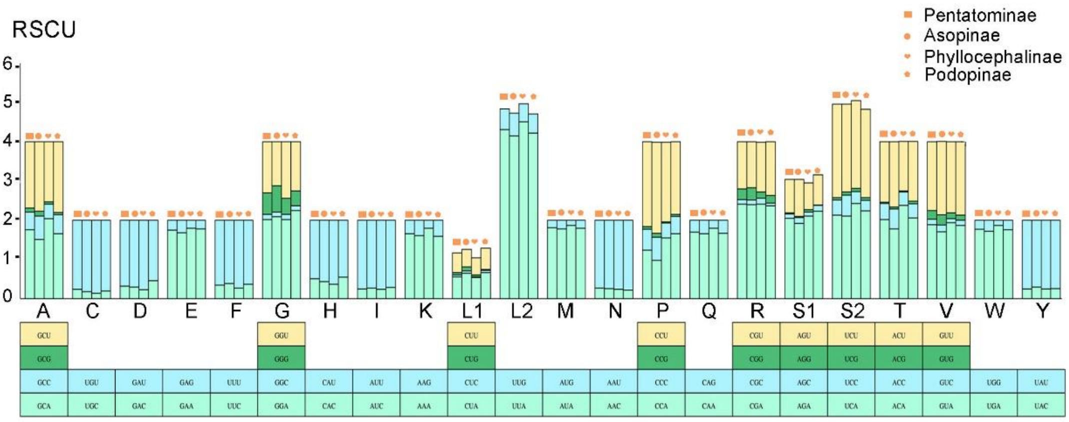

Supplementary Figure 1: RSCU of the mitochondrial genome of four subfamilies of Pentatomidae.

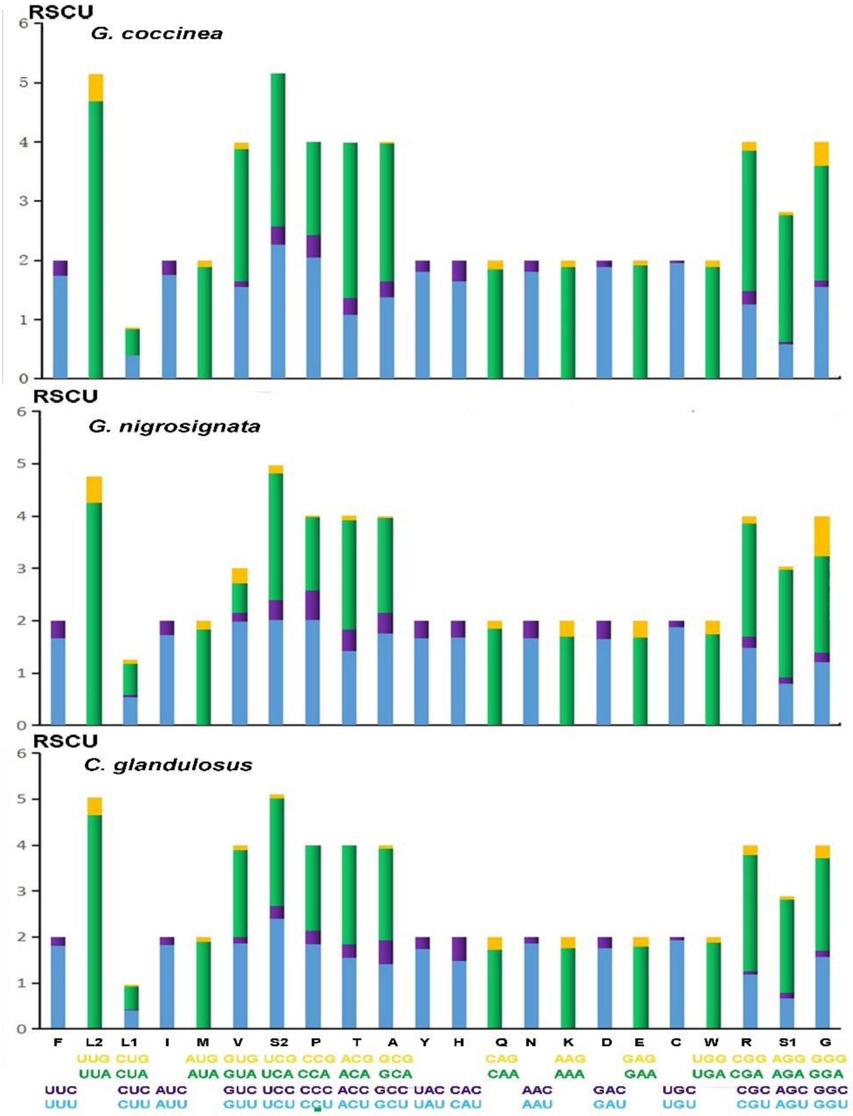

Supplementary Figure 2: RSCU of the mitochondrial genome of three species of the Phyllocephalini.

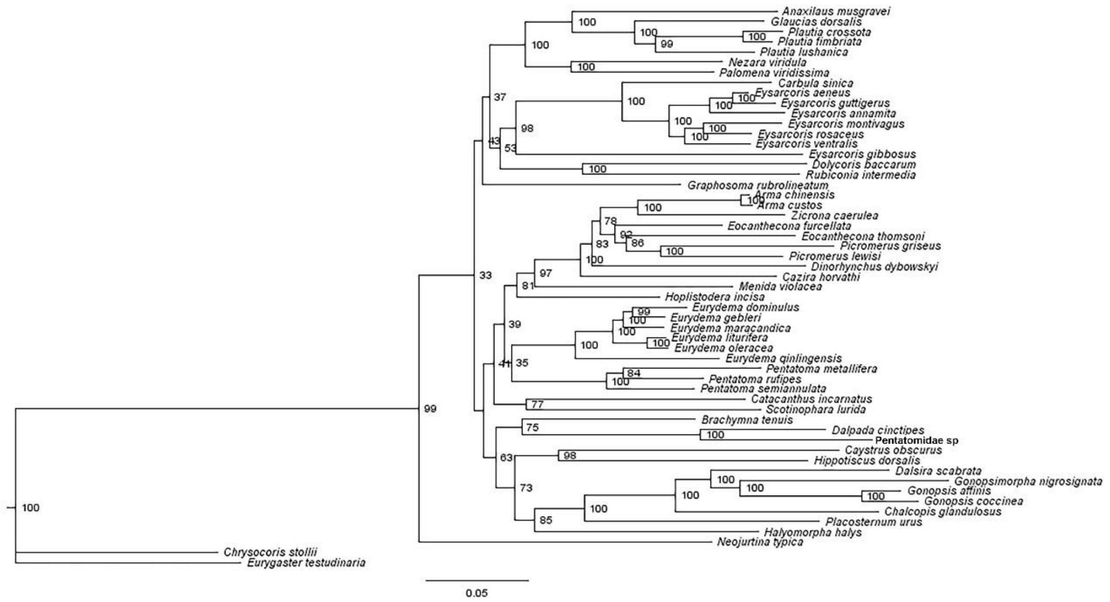

Supplementary Figure 3: The phylogenetic tree of Pentatomidae was constructed from DNA sequences of 13 PCGs using ML method. Numbers on branches are bootstrap (BS).

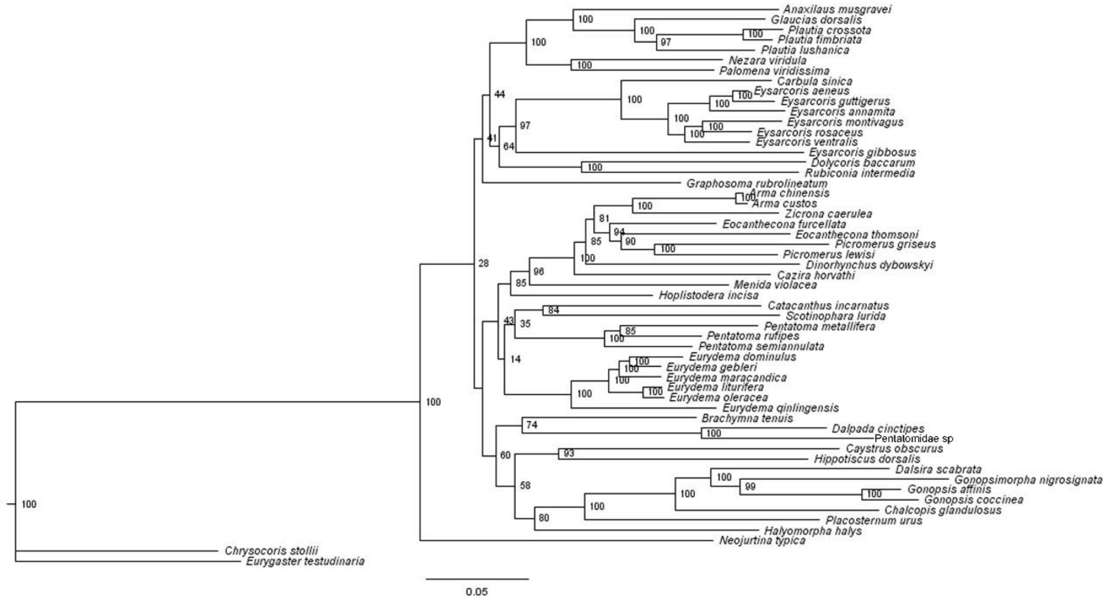

Supplementary Figure 4: The phylogenetic tree of Pentatomidae was constructed from DNA sequences of 13 PCGs excluding the third site by ML method. Numbers on branches are bootstrap (BS).
